# Variation in DNA methylation patterns of grapevine somaclones (*Vitis vinifera L*.)

**DOI:** 10.1186/1471-2229-8-78

**Published:** 2008-07-15

**Authors:** Paul Schellenbaum, Volker Mohler, Gerhard Wenzel, Bernard Walter

**Affiliations:** 1 Université de Haute Alsace, Laboratoire Vigne Biotechnologies & Environnement, 33 rue de Herrlisheim, BP 50568, F-68008 Colmar, France; 2 Technische Universität München, Lehrstuhl für Pflanzenzüchtung, Am Hochanger 2, D-85350 Freising-Weihenstephan, Germany; 3 Bavarian State Research Centre for Agriculture, Institute for Crop Science and Plant Breeding, Am Gereuth 8, D-85354 Freising, Germany

## Abstract

**Background:**

In traditional vine areas, the production should present a typicity that partly depends on the grapevine variety. Therefore, vine improvement is considered difficult because of the limited choice in the natural variability of the cultivars within the limits of their characteristics. A possibility to circumvent this problem is the use of somatic variability. *In vitro *somatic embryogenesis and organogenesis can lead to genotypic and phenotypic variations, described as somaclonal variation, that could be useful for the selection of improved grapevine genotypes.

**Results:**

In order to study tissue culture-induced variation of grapevine, we have analysed 78 somaclones obtained from somatic embryos of two distinct cultivars using molecular marker techniques. SSRs were only useful to verify the conservation of the microsatellite genotype between the somaclones and the respective mother clones. AFLP polymorphism between mother clones and somaclones was 1.3–2.8 times higher to that found between clones. However, a majority of the somaclones (45/78) exhibited only few changes. Seven and five somaclones of 'Chardonnay 96' and 'Syrah 174', respectively, which covered at least all polymorphic loci found in AFLP analysis were used for MSAP study. All of the 120 polymorphic fragments were found only in the somaclones. The percentage of full methylation at CCGG recognition sites was slightly higher in somaclones due to more polymorphic bands generated after cleavage by *Eco*RI/*Hpa*II. Different digestion patterns revealed different methylation status, especially different levels of de-methylation, that are the consequence of the *in vitro *culture.

**Conclusion:**

MSAP highlights DNA methylation variation in somaclones compared to mother clones and, therefore, is a powerful tool for genotypic characterisation of somatic embryo-derived grapevines. The detection of the same polymorphic bands in numerous somaclones of different cultivars suggests the possibility of hot spots of DNA methylation variation. SSR profiles of the 'Chardonnay' and 'Syrah' somaclones were the same as of the respective mother clones. The somaclones exhibited a higher AFLP variation than clones obtained via traditional clonal selection in the field. Therefore, somatic embryogenesis through *in vitro *culture technique could be useful for the selection of improved cultivars with subtle changes but conserving their main characteristics.

## Background

The grapevine is economically the most important cultivated fruit crop in the world. The genus *Vitis *L. comprises 40 to 60 Asian species, about 25 from North America and a single European species, *Vitis vinifera *L. The latter species is grown for the production of high quality fruits and wines, but shows sensitivity to many pathogens (phylloxera, downy and powdery mildew, rots, etc.). The former species are used mostly for breeding rootstocks and fungus-resistant scion cultivars. Grapevines are propagated by cuttings and the resulting clones are genetically identical to each other (except for somatic mutations) and to the mother plant (the original seedling from which cultivars were derived).

Clonal variability within *V. vinifera *cultivars (cépages) has been used in traditional viticultural areas where 'trueness-to-type' is essential and even mandatory. But this clonal (pomological and sanitary) selection often remains limited and empirical. One way to broaden clonal selection could be by the induction of somaclonal variations which ranged from easy to detect deviations in general morphological characteristics to subtle deviations in e.g., vigour, bunch and berry sizes, sugar and acid concentrations, and flavour components. Desperrier *et al*. [1] analysed 13 *V. vinifera *'Gamay' somaclones over a period of ten years: all the somaclones were constantly less fertile and less productive in comparison with the vegetatively *ex vitro*-propagated control. This resulted in a sharp increase in sugar content and a better maturity. The authors concluded from the differences observed between the somaclones that the expected variability is present. There are few reports, however, of such extensive agronomic assays of grapevine somaclones and little is known about the mechanisms involved. Somaclonal variation caused by *in vitro *culture is also called tissue culture-induced variation [2,3]. Somaclonal variation may involve chromosome number and structure, gene mutation, altered sequence copy number, activation of transposable elements, somatic crossing-over, sister chromatide exchange, DNA amplification and deletion, and change in methylation pattern [4,5]. Genotype and *in vitro *culture conditions (type of explants, medium, duration) influence the occurrence and frequency of somaclonal variation [6]. Environmental stresses induce genetic and epigenetic changes that trigger DNA methylation. DNA methylation can generate novel and heritable phenotypic variations [7]. Cytosine methylation of DNA in plants occurs at CpG, CpNpG (where N is any nucleotide), and asymmetric CpHpH sites (where H is adenine, cytosine or thymine). Cytosine methylation polymorphism is greater than DNA polymorphism in rice [8], *Arabidopsis thaliana *[9] or cotton [10].

Methylation patterns were reported to vary among *in vitro*-regenerated plants and their progeny e.g. in rice [11], corn [12,13], oil palm [14], banana [15], *Medicago truncatula *[16], rose [17], hop [18], barley [19,20], *Codonopsis lanceolata *[21] and potato [22].

In this work, we studied the somaclonal variation of a great number of grapevine somatic plants from two different *V. vinifera *cultivars. Six SSRs markers were used to assess the conformity of the somaclones to the mother clones. We focused on possible changes in the methylation pattern of grapevine somaclones, by using a methylation sensitive AFLP. Different methylation states of specific loci were detected only in the somaclones by comparison to the mother clones. The detected somaclonal variations suggest that DNA de-methylation occurred during the *in vitro *culture process.

## Results

### Microsatellite analysis

DNA was extracted from young leaves of greenhouse grown *V. vinifera *'Chardonnay' clones 96, 131 and 548 and 'Syrah' clones 174 and 'd'Auvergne', 56 somaclones derived from 'Chardonnay 96' and 22 somaclones from 'Syrah 174', and other *V. vinifera *cultivars but also inter-specific hybrids (Table 1). DNA was analysed at six microsatellite loci: VMC6C10, VMC5G7, VVMD5, VVMD7, VVMD27 and VVS2 which were mapped to linkage groups 14, 2, 16, 7, 5 and 11, respectively, on the integrated genetic map of grapevine [23]. The reference cultivars showed microsatellite allele sizes consistent with those described in the literature [24] and, for a given cultivar ('Chardonnay' or 'Syrah'), clones did not show any difference in the microsatellite profile. In all cases, somaclones and the respective mother clones shared the same alleles at all six marker loci (Table 1).

**Table 1:** SSR allele sizes (in base pairs) at 6 loci in somaclones and mother clones of *V. vinifera *'Syrah 174' and 'Chardonnay 96' and 12 *Vitis *accessions.

**Microsatellite locus**		**VVS2**	**VVMD5**	**VVMD7**
Controls	Syrah 174	133(BA1)	226(CF1)/232(TR1)	240(CF1)
	Syrah d'Auvergne	133(BA1)	226(CF1)/232(TR1)	240(CF1)
Somaclones	Syrah 174	133(BA1)	226(CF1)/232(TR1)	240(CF1)

Controls	Chardonnay 131	137(CH1)/143(CH2)	234(CH1)/238(CH2)	240(CF1)/244(TR1)
	Chardonnay 548	137(CH1)/143(CH2)	234(CH1)/238(CH2)	240(CF1)/244(TR1)
	Chardonnay 96	137(CH1)/143(CH2)	234(CH1)/238(CH2)	240(CF1)/244(TR1)
Somaclones	Chardonnay 96	137(CH1)/143(CH2)	234(CH1)/238(CH2)	240(CF1)/244(TR1)

Controls	Pinot Noir 743	137(CH1)/151(SI1)	228(MU1)/238(CH2)	240(CF1)/244(TR1)
	Sangiovese	133(BA1)	226(CF1)/236(MU2)	240(CF1)/262(99R2)
	Furmint	133(BA1)/153(SI2)	226(CF1)/240(CF2)	240(CF1)/250(MU2)
	Sauvignon 159	133(BA1)/151(SI1)	228(MU1)/232(TR1)	240(CF1)/256(PO2)
	Sauvignon 530	133(BA1)/151(SI1)	228(MU1)/232(TR1)	240(CF1)/256(PO2)
	101-14 clone 1043	133(BA1)/143(CH2)	256(1MG1)/266(1MG2)	244(TR1)/252(FE2)
	*V. riparia *1030	141(GO2)/145(SU1)	266(1MG2)	252(FE2)/264(CF2)
	Seibel 9110	133(BA1)	226(CF1)/252(33C1)	246(33C1)/252(FE2)
	41B clone 194	135(BA2)/143(CH2)	226(CF1)	232(FE1)/240(CF1)

				

**Microsatellite locus**		**VVMD27**	**VMC5G7**	**VMC6C10**

Controls	Syrah 174	189(CS2)/191(ME2)	196/214	126/130
	Syrah d'Auvergne	189(CS2)/191(ME2)	196/214	126/130
Somaclones	Syrah 174	189(CS2)/191(ME2)	196/214	126/130

Controls	Chardonnay 131	181(CF1)/189(CS2)	196/220	114/140
	Chardonnay 548	181(CF1)/189(CS2)	196/220	114/140
	Chardonnay 96	181(CF1)/189(CS2)	196/220	114/140
Somaclones	Chardonnay 96	181(CF1)/189(CS2)	196/220	114/140

Controls	Pinot Noir 743	185(PI1)/189(CS2)	190/216	114/130
	Sangiovese	179(MU1)/185(PI1)	202/218	130
	Furmint	179(MU1)/194(MU2)	196/214	130/140
	Sauvignon 159	175(CS1)/189(CS2)	196/214	114/140
	Sauvignon 530	175(CS1)/189(CS2)	196/214	114/140
	101-14 clone 1043	197(1MG1)/205(4MA1)	182/184	126/130
	*V. riparia *1030	207(1MG2)/211(16C2)	182/184	128
	Seibel 9110	179(MU1)/185(PI1)	212/214	126/130
	41B clone 194	189(CS2)/191(ME2)	186/214	122

Reference alleles of respective SSR loci according to This *et al*. [24] are given in brackets.

Overall, we detected a total of 52 alleles with the six microsatellite markers in the panel of grapevine genotypes investigated. No novel microsatellite alleles different from those published were detected either in the various *V. vinifera *varieties and inter-specific hybrid rootstock cultivars or in other *Vitis *species.

### AFLP analysis

**Figure 1:**
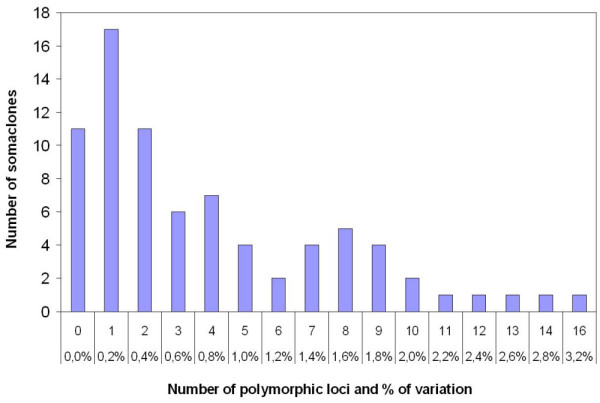
**Distribution of polymorphic AFLP (*Eco*RI/*Msp*I) loci in *V. vinifera *somaclones**. Numbers of somaclones depending on the numbers of polymorphic AFLP loci are presented. Percentages of variation (number of polymorphic loci/total number of detected loci) are given.

Using five *Eco*RI/*Msp*I primer combinations, *V. vinifera *'Chardonnay 96' and 'Syrah 174' which were analysed in three biological replications, yielded an average of 101 fragments per primer combination, totalling to 505 studied loci. DNA profiles were then generated for the 56 'Chardonnay 96' and 22 'Syrah 174' somaclones and compared to their mother clones. In this comparison, 25 and 21 loci were found to show polymorphism for the somaclones of 'Chardonnay 96' and 'Syrah 174', respectively (Table 2). This corresponds to 5.0% and 4.2% of the total variation present in 'Chardonnay 96' and 'Syrah 174' somaclones, respectively. Ten 'Chardonnay 96' and one 'Syrah 174' somaclones did not show any polymorphism with the five primer combinations used (Figure 1). For the remaining somaclones the number of polymorphic fragments ranged between one (0.2% of variation) and 16 (3.2%), with an average of 4.5 polymorphisms per plant. All these fragments were novel bands as they were found only in the somaclones but not in the mother clones. No loss of original bands present in the mother clones was observed. In addition, 12 of these polymorphic loci were common to both 'Chardonnay 96' and 'Syrah 174' somaclones. The number of somaclones showing the same variable marker ranged between 2 and 34, as shown for the polymorphic fragment E46M84-358 (Table 2). Only 3 singletons, i.e., variant fragments that occurred only once in the somaclones were found. Summarised, out of 39,390 DNA fragments amplified in 78 *V. vinifera *somaclones, 307 showed polymorphism.

**Table 2:** Total number of somaclones derived from *V. vinifera *variable for each polymorphic AFLP (*Eco*RI/*Msp*I) locus.

Primers	'Chardonnay 96'		'Syrah 174'	
		
	Locus (size in bp)	Numbers of somaclones	Locus (size in bp)	Numbers of somaclones
E32HM35	**62**	**1**	**62**	**1**
	253	9	**373**	**2**
	300	6		
	344	1		
	**373**	**7**		
	473	2		

E33HM46	251	2	**343**	**5**
	**343**	**15**	**352**	**6**
	**352**	**4**	365	1
			372	2

E42HM84	301	6	200	3
	**303**	**18**	258	2
	476	8	**303**	**3**
	**488**	**6**	405	8
	**490**	**5**	480	6
			**488**	**1**
			**490**	**3**

E45HM34	106	13	80	10
	**212**	**18**	155	1
	**242**	**8**	**212**	**9**
	252	7	**242**	**5**
	383	3	430	5
	484	4		

E46HM84	209	27	**333**	**4**
	**333**	**4**	**358**	**7**
	354	2	**384**	**7**
	**358**	**27**		
	**384**	**12**		

Polymorphic bands present in somaclones irrespective of cultivar origin are given in bold and unique fragments are underlined.

The percentage of total DNA variation was 1.8 (9 variable markers/505 total markers) for the 3 different Chardonnay clones '96', '131' and '548', and 3.2 (16/505) for the comparison of 'Syrah 174' and 'Syrah d'Auvergne', a selection of 'Syrah' grapevine multiplied starting from old stocks found in the Auvergne region. At the level of *V. vinifera *cultivars, the degree of polymorphism between 'Chardonnay 96' and 'Syrah 174' accounted for 31.9% (161/505).

### MSAP analysis

**Figure 2:**
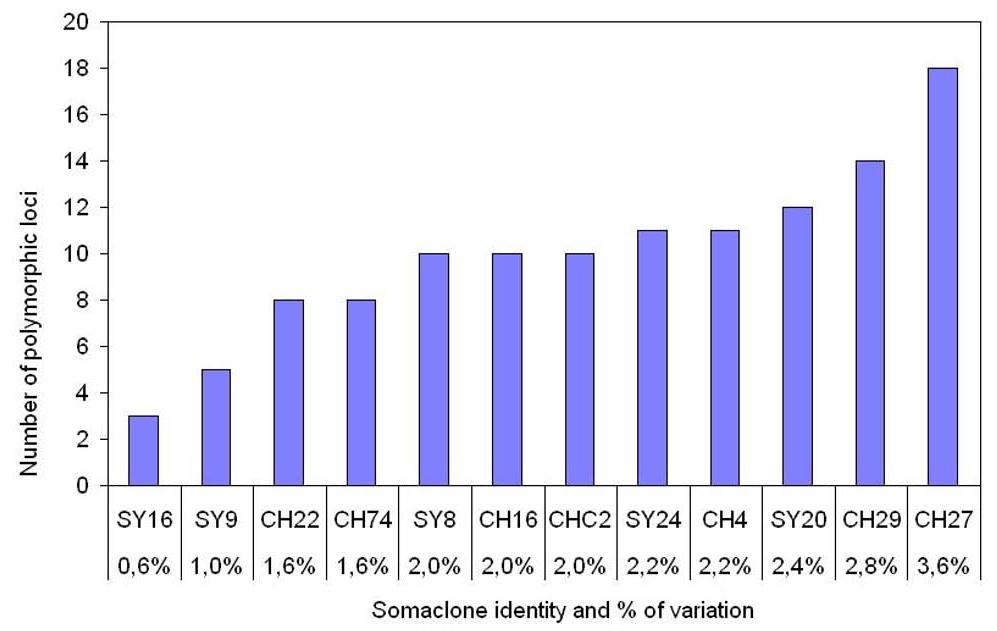
**Distribution of polymorphic MSAP (*Eco*RI/*Msp*I and *Eco*RI/*Hpa*II) loci in 12 *V. vinifera *somaclones**. Numbers of polymorphic MSAP loci in 12 selected 'Syrah' (SY) and 'Chardonnay' (CH) somaclones. Percentages of variation (number of polymorphic loci/total number of detected loci) are given.

**Figure 3:**
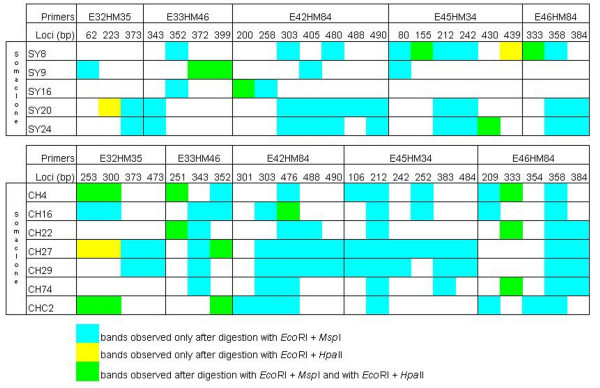
**MSAP patterns (*Eco*RI/*Msp*I and *Eco*RI/*Hpa*II) of 12 *V. vinifera *somaclones**. Polymorphic MSAP fragments (sizes in bp) detected in 5 'Syrah' (SY) and 7 'Chardonnay' (CH) somaclones.

Seven and five somaclones of 'Chardonnay 96' and 'Syrah 174', respectively, which covered at least all polymorphic loci found in the *Eco*RI/*Msp*I fragment analysis were chosen for digestion with the isoschizomer *Hpa*II. For estimation of methylation degree, marker profiles obtained from *Eco*RI/*Msp*I and *Eco*RI/*Hpa*II digested DNA from corresponding somaclones were compared, and 120 differentially amplified fragments were observed. The numbers of variable markers per somaclone ranged between 3 (Syrah somaclone no. 16) and 18 (Chardonnay somaclone no 27) corresponding to 0.6 and 3.6% of variation, respectively (Figure 2). As for AFLP analysis, all polymorphisms appeared as novel fragments in the somaclones. These fragments were generated after differential recognition of the two isoschizomers, producing different MSAP patterns in the 12 somaclones (Figure 3). Ninety-eight (82%) fragments were produced from cleavage by *Msp*I but not *Hpa*II indicating full methylation of the internal but not of the external cytosine of recognition sequences in somaclones. Only 4 fragments (3%) resulted from cleavage by *Hpa*II but not *Msp*I due to the hemi-methylation of external cytosines but no methylation of the internal cytosines. These MSAP loci correspond to novel bands compared to AFLP analysis. Eighteen fragments (15%) arose from cleavage by both restriction enzymes indicating full de-methylation of both cytosines in the DNA of the somaclones compared to the mother clones. Interestingly, the occurrence of the 2 variable markers E32M35-253 and E32M35-300 in 4 'Chardonnay 96' somaclones was due to the three different digestion patterns, indicating different methylation states of the respective somaclones at these marker loci. In addition, 11 polymorphic MSAP loci were common between the 12 'Chardonnay' and 'Syrah' somaclones. The numbers of grapevine somaclones for each variable band are shown in Table 3.

**Table 3:** Polymorphic MSAP bands present in somaclones irrespective of cultivar origin.

Primers	Locus (size in bp)	Syrah	Chardonnay	Total
E32HM35	373	2	2	4

E33HM46	343	2	5	7
	352	2	4	6

E42HM84	303	3	5	8
	488	1	3	4
	490	2	2	4

E45HM34	212	3	7	10
	242	3	2	5

E46HM84	333	1	3	4
	358	3	7	10
	384	2	4	6

Based on these MSAP profiles, the numbers of non-methylated, hemi-methylated and fully-methylated CCGG sites were calculated (Table 4). In the mother clones 'Syrah' and 'Chardonnay', 10.1% and 12.3% of the target sequences, respectively, were fully methylated at the internal cytosines, whereas hemi-methylation at the external cytosines was observed for 2.4% and 4.2%, respectively. All 12 somaclones showed slightly higher level in full methylation of the internal Cs (10.4% to 12.0% for 'Syrah' somaclones, 13.2% to 14.7% for 'Chardonnay' somaclones). Nonetheless, these modifications are not statistically significant (Khi2 test; P_(2-tailed) _= 0.208 > 0.05).

**Table 4:** Number of bands amplified by MSAP in 12 *V. vinifera *somaclones and in the mother clones 'Syrah 174' (SYT) and 'Chardonnay 96' (CHT).

Plant	Total bands	Non-methylated CCGG sites (%)	Methylated CCGG sites		
			
			Fully-methylated sites (%); internal C	Hemi-methylated sites (%); external C	Total (%)
SYT	504	441 (87.5)	51 (10.1)	12 (2.4)	63 (12.5)
SY8	514	443 (86.2)	58 (11.3)	13 (2.5)	71 (13.8)
SY9	509	443 (87.0)	54 (10.6)	12 (2.4)	66 (13.0)
SY16	507	442 (87.2)	53 (10.4)	12 (2.4)	65 (12.8)
SY20	516	441 (85.5)	62 (12.0)	13 (2.5)	75 (14.5)
SY24	515	442 (85.8)	61 (11.9)	12 (2.3)	73 (14.2)

CHT	506	423 (83.5)	62 (12.3)	21 (4.2)	83 (16.5)
CH4	517	427 (82.6)	69 (13.3)	21 (4.1)	90 (17.4)
CH16	516	424 (82.2)	71 (13.7)	21 (4.1)	92 (17.8)
CH22	514	425 (82.7)	68 (13.2)	21 (4.1)	89 (17.3)
CH27	524	424 (80.9)	77 (14.7)	23 (4.4)	100 (19.1)
CH29	520	423 (81.4)	76 (14.6)	21 (4.0)	97 (18.6)
CH74	514	424 (82.5)	69 (13.4)	21 (4.1)	90 (17.5)
CHC2	516	426 (82.5)	69 (13.4)	21 (4.1)	90 (17.5)

## Discussion

Tissue culture-induced changes, including morphological, cytological, biochemical and genetic/epigenetic alterations, have been frequently reported. However, the mechanism underlying this so called somaclonal variation remains largely unclear [3,18]. Modifications in cytosine methylation was detected in many studies indicating that epigenetic alterations may play an important role [11-22]. Recent works pointed out the possible interactions of both genetic and epigenetic changes induced by the plant tissue culture process [20,21].

In this study, SSRs, AFLP and MSAP techniques were applied to assess the genetic and epigenetic stability of 56 'Chardonnay 96' and 22 'Syrah 174' somaclones.

SSRs markers are very helpful to distinguish grapevine cultivars. From a comparative study in ten laboratories [24] as few as two markers turned out to be sufficient to differentiate each of 46 cultivars. Four additional loci were used in order to increase polymorphism and thus reduce the probability of false identification. Using 6 microsatellites, among which four from the work of This *et al*. [24], we did not detect any difference between mother clones and somaclones. Therefore, the *in vitro *culture-derived plants present genotypic conformity to the cultivar, and phenotypic trueness-to-type will be further confirmed by observations in a field plot recently planted.

Imazio *et al*. [25] showed that SSRs were not a powerful tool for clonal distinction of *V. vinifera *'Traminer'. Microsatellite analysis on 25 *V. vinifera *'Sangiovese' accessions carried out at 8 loci did not show polymorphism, except for 3 accessions. Previous traditional ampelometric studies already suggested that these 3 divergent accessions were not really 'Sangiovese' [26]. Thus, microsatellites are not helpful neither for the detection of clones for a specific grapevine cultivar nor, as has been shown in our study, for the detection of somaclonal variation in *V*.*vinifera*.

In contrast, using AFLP and MSAP techniques Imazio *et al*. [25] could distinguish 16 out of 24 examined 'Traminer' clones, though the average similarity was high (97.1%). In addition, AFLP markers were successfully applied to differentiate a grape sport of 'Flame Seedless' displaying earlier bud burst from its parental genotype [27].

In our AFLP study, 25 and 21 loci showed polymorphism in 'Chardonnay' and 'Syrah' somaclones, respectively, corresponding to a genetic variation frequency of 5% and 4.2%. In regenerants obtained by tissue culture of wild barley, the genetic variation frequency was higher (9.3%) and the majority of polymorphic bands were losses of original bands [20]. No losses of bands were scored in our study what might explain the lower level of variation observed. Few somaclones accumulated a large number of polymorphic bands. Only 2 'Syrah 174' and 5 'Chardonnay 96' somaclones showed ten or more variant bands, whereas the majority (45/78) exhibited less than 4 polymorphic bands. This distribution was also observed in tissue culture of wild barley, where a small number of regenerants accumulated a high number of variant bands [20].

Another possibility to investigate somaclonal variation is to evaluate the degree of DNA methylation. Studies of both global methylation levels and methylation of specific sites showed that the variation in the DNA methylation occurs frequently in the *in vitro *culture process. The majority of the changes are decreases in methylation, at a frequency which is three times or more higher than that of the increases [3,19]. In callus-derived hop plants, 83% changes of the polymorphic loci detected by MSAP between controls and regenerated hop somaclones were de-methylation of the recognition sites. Increase in the variation was observed in prolonged callus culture [19]. Some somatic embryo-derived oil palms showed a 'mantled' variant phenotype that affected the formation of floral organs in both male and female flowers. A deficit in DNA methylation was measured in regenerants obtained through somatic embryogenesis, however, it was not possible to correlate it with the occurrence of the aberrant phenotype [28,29]. In contrast, for Bamboo somatic embryos, no epigenetic changes could be detected by MSAP analysis of three samples from different stages of *in vitro *culture, using three primer pairs [30].

Using 5 primer pairs, we obtained 120 polymorphic MSAP fragments, among which 95% were similar to those reported in the AFLP analysis. Therefore, polymorphic bands were in large part due to variation in methylation, however, mutations should not be excluded. In a study of tissue culture-induced variation in barley, the average level of variation was 6%, and about 1.7% were attributed to nucleotide mutations whereas the remainder were changes in methylation state [19]. Thus, some of the detected variations in the grapevine somaclones could perhaps also arise from a nucleotide mutation. Tissue culture can also uncover somatic mutations that accumulate in grapevine [31,32] and could also explain some of the detected polymorphism.

The cytosine methylation level in all 12 somaclones was slightly higher compared to the level of mother clones (Table 4). This was mainly due to a higher level in full methylation of the internal Cs as most of the polymorphic bands were produced from cleavage by *Msp*I but not *Hpa*II. In the case of tissue culture in wild barley, Li *et al*. reported a significant decrease in cytosine methylation levels at the CCGG sites [20]. From 10 regenerants of *Codonopsis lanceolata*, 7 showed little increase in full methylation at internal cytosines, but the total methylation level appeared largely stable. However, as it is the case in our study, the authors reported that none of the alterations of cytosine methylation levels were statistically significant [21]. In both studies, MSAP analysis revealed alterations of methylation patterns at different loci and the regenerants were distinct from each other and from the donor plants [20,21]. In our study, all the 12 somaclones showed different MSAP profiles and differed from the respective mother clone (Figure 3), suggesting also an extensive epigenetic diversification in *V. vinifera *somaclones.

No losses of bands were observed in the somaclones compared to the mother clones, indicating that all monomorphic MSAP loci between the mother clones and somaclones showed the same degree of cytosine methylation. These loci were not affected by the *in vitro *culture process. New bands were detected only in somaclones that could arise from a modification of the cytosine methylation status of the CCGG recognition sites in these plants. De-methylation of one or more cytosine(s) could produce a new MSAP fragment not detected in mother clones. An argument for this hypothesis, is the fact that the 3 different digestion patterns, i.e. fragments generated only by *Eco*RI/*Msp*I, only by *Eco*RI/*Hpa*II or by both couples of restriction enzymes, were found for E32M35-253 and E32M35-300 in 4 different 'Chardonnay 96' somaclones. The result indicates that these marker loci display different methylation states. In mother clone 'Chardonnay' both internal and external cytosines of recognition sequences could be methylated, as well as on both strands (no band detected); in somaclones, de-methylation of different cytosines could have occurred leading to differential cleavage by *Msp*I and/or *HpaII*, resulting in different MSAP patterns. Sequencing of these particular fragments in somaclones and mother clones should help to resolve this question.

Altogether, our MSAP analysis suggests modification in the level of cytosine methylation and alterations in DNA methylation patterns, particularly de-methylation, during *in vitro *culture.

RAPD analysis of rye somatic embryos revealed hot spots of DNA instability as the same polymorphic band varied in several plants obtained from different calli [33]. In a study of the tissue-culture induced variation in barley, some of the possible methylation patterns were not identified, and some others were very rare, supporting a non-random induction of (epi)mutations. Interestingly, variation in methylation status was affected by these non-random events rather than being sequence modifications [19]. Our AFLP and MSAP analyses of grapevine somatic embryos also showed that some of the polymorphic bands are present in many somatic embryos irrespective of cultivar origin ('Chardonnay' or 'Syrah'). These bands could originate from hypervariable regions in the grapevine genome and perhaps reveal hot spots of DNA methylation changes at least during somatic embryogenesis.

## Conclusion

For micropropagation and genetic transformation it is necessary that plants regenerated from callus culture are genetically stable without any significant phenotypic variation. Trueness-to-type is essential for the grapevine, especially in traditional vine areas where high quality is a prerequisite. On the other hand, somaclones regenerated from callus cultures possibly may be a source of variation with potential applications in plant breeding.

By using SSRs, AFLPs and MSAPs, we have analysed 78 somaclones obtained from two distinct grapevine cultivars to determine the level of somaclonal variation. SSRs were only useful to verify the conservation of the microsatellite genotype of the somaclones as to their corresponding mother clones. AFLP polymorphism between mother clones and somaclones was 1.3–2.8 times higher to that found between clones. MSAP is a very powerful method to highlight DNA methylation variation in somaclones compared to mother clones. Different digestion patterns revealed different methylation status, especially different levels of de-methylation, that are the consequence of the *in vitro *culture.

Moreover, due to the detection of the same polymorphic bands in numerous somaclones of different cultivars, we presume the possibility of hot spots of DNA methylation. Further studies are needed to evaluate this supposition and to better understand epigenetic control during somatic embryogenesis and plant development.

As the degree of variation is higher to that of clonal selection, somatic embryogenesis could be a useful technique for the selection of improved cultivars with subtle changes, but conserving their main characteristics. Nevertheless, trueness-to-type has to be confirmed by phenotypic observations and wine testing.

## Methods

### Plant materials

Fifty-seven somaclones were obtained from *Vitis vinifera *cv. 'Chardonnay clone 96' and 22 somaclones from *V*.*vinifera *cv. 'Syrah clone 174' as described elsewhere [34]. As controls in molecular marker analyses, we used *V*.*vinifera *'Chardonnay clone 96' and 'Syrah clone 174', 'Chardonnay clone 131', 'Chardonnay clone 548', a distinct Syrah obtained from old stocks grown in the Auvergne region designated as 'Syrah d'Auvergne', 'Furmint', 'Sauvignon clone 530', 'Sauvignon clone 159', 'Sangiovese', and 'Pinot Noir clone 743'. As controls distinct from *V. vinifera*, we used *V. riparia *'Gloire de Montpellier clone 1030', inter-specific hybrids: *V. riparia *× *V. rupestris *'101-14 clone 1043', Seibel 9110 = Verdelet ('Seibel 5455 × Seibel 4938') and the grapevine rootstock '41B clone 194' (*V. vinifera *'Chasselas' × *V. berlandieri*). Plants were grown in a greenhouse under controlled conditions. Fresh unexpanded young leaves were collected and kept at -80°C until used for DNA extraction.

### DNA extraction

About 80 mg of leaves were ground in liquid nitrogen using a grinder (Retsch MM200, Haan, Germany) and total DNA was extracted with the QIAGEN DNeasy Plant Mini Kit (Qiagen, Hilden, Germany) as described by the supplier. AP1 lysis buffer was supplemented with 2.5% PVP40. DNA quality and concentration were checked by electrophoresis in 1% agarose gels.

### Microsatellite analysis

Six different genomic microsatellite loci were analysed: VVS2, VVMD5, VVMD7, VVMD27 [24], VMC6C10 and VMC5G7 (Vitis Microsatellites Consortium, Dr. Rosa Arroyo Garcia and Dr. Kirsten Wolff). Amplification reactions were performed in a total volume of 25 μl consisting of 10 to 20 ng template DNA, 10 ng forward primer labelled either with 6-FAM or HEX fluorophore, 10 ng non-labelled reverse primer, 200 μM of each dNTP (MP Biomedicals, Heidelberg, Germany), 1× PCR Buffer and 0.5 unit *Taq *DNA Polymerase (MP Biomedicals). PCR was carried out in a MJ Research PTC 200 Thermal Cycler (Waltham, MA, USA).

The cycling program consisted of the following steps: 2 min at 94°C followed by 35 cycles of 45 s at 94°C, 30 s at 52°C and 1 min at 72°C and a final extension step of 7 min at 72°C. The amplification products were detected with an ABI PRISM^® ^377 DNA Sequencer (Applied Biosystems, Darmstadt, Germany) using 5% denaturing polyacrylamide gels (36 cm) and GeneScan-500 TAMRA as internal size standard. The sizes of DNA fragments were determined using GeneScan™ analysis software version 3.1 (Applied Biosystems).

### AFLP and MSAP analysis

DNA (200 – 400 ng) was digested with 5U *Eco*RI and 5 U *Msp*I or *Hpa*II (New England BioLabs, Frankfurt am Main, Germany) in a final volume of 30 μl containing 1× NEB2 buffer and BSA (75 ng/μl). DNA fragments were concurrently ligated to *Msp*I-*Hpa*II (50 pmol) and *Eco*RI (5 pmol) adapters for 3 h at 37°C using 1 mM ATP and 1U T4 DNA ligase (New England BioLabs). Samples were diluted with TE_0.1_buffer to a final volume of 200 μl. The sequences of *Eco*RI and *Msp*I/*Hpa*II adapters were those described by Vos *et al*. [35] and Xiong *et al*. [36], respectively.

Pre-amplification was performed in a mixture containing 4 μl of the above reaction, 2.5 pmol of each E01 and HM0 primers (Table 5), 200 μM dNTPs (MP Biomedicals), 1× PCR buffer and 0.5 U *Taq *DNA Polymerase (MP Biomedicals) of a final volume of 20 μl. After 2 min each at 65°C and 94°C, pre-amplification was carried out for 20 cycles of denaturation (20 s at 94°C), annealing (30 s at 56°C) and extension (2 min at 72°C). After a final elongation step (2 min at 72°C and 30 min at 60°C), the pre-amplification product was diluted 1:10 in TE_0.1 _buffer.

**Table 5:** AFLP and MSAP primer sequences.

Pre-amplification	
E01 5'-GACTGCGTACCAATTCA-3'	HM0 5'-ATCATGAGTCCTGCTCGG-3'

Selective amplification	

E32 5'-GACTGCGTACCAATTCAAC-3'	HM35 5'-ATCATGAGTCCTGCTCGGACA-3'
E33 5'-GACTGCGTACCAATTCAAG-3'	HM46 5'-ATCATGAGTCCTGCTCGGATT-3'
E42 5'-GACTGCGTACCAATTCAGT-3'	HM84 5'-ATCATGAGTCCTGCTCGGTCC-3'
E45 5'-GACTGCGTACCAATTCATG-3'	HM34 5'-ATCATGAGTCCTGCTCGGAAT-3'
E46 5'-GACTGCGTACCAATTCATT-3'	HM84 5'-ATCATGAGTCCTGCTCGGTCC-3'

Selective amplification was carried out using selective primer combinations as described in Table 5. Reactions were performed in a total volume of 20 μl using 4 μl of the pre-amplification mixture, 1.5 pmol of *Eco*RI primer fluorescence dye-labelled either with 6-FAM or JOE fluorophore, 5 pmol of *Msp*I-*Hpa*II primer, 200 μM dNTPs (MP Biomedicals), 1× PCR buffer and 0.5 U *Taq *DNA Polymerase (MP Biomedicals). Touch down PCR was performed as described by Vos *et al*. [35].

Electrophoresis platform and conditions used for AFLP analysis were the same as for microsatellites but using GeneScan-500 ROX as internal size standard. Sizes of DNA fragments were determined using GeneScan™ analysis software version 3.1 and polymorphisms were scored using Genotyper™ DNA fragment analysis software version 2.5.2 (Applied Biosystems).

## Authors' contributions

PS prepared DNA materials for analysis, carried out the molecular studies and scored gels, quantified the polymorphisms, as well as participated in writing the manuscript. VM participated in the design of the study, in scoring and quantification of polymorphisms, and drafted the manuscript. GW participated in the conceptual work and the drafting of the manuscript. BW conceived the study, participated in its coordination and helped to draft the manuscript. All authors read and approved the final manuscript.
